# Characterization and Analysis of Strain Heterogeneity at Grain-Scale of Titanium Alloy with Tri-Modal Microstructure during Tensile Deformation

**DOI:** 10.3390/ma11112194

**Published:** 2018-11-06

**Authors:** Pengfei Gao, Yanxi Li, Ronghai Wu, Zhenni Lei, Yang Cai, Mei Zhan

**Affiliations:** 1 State Key Laboratory of Solidification Processing, Shaanxi Key Laboratory of High-Performance Precision Forming Technology and Equipment, School of Materials Science and Engineering, Northwestern Polytechnical University, Xi’an 710072, China; liyanxinwpu@163.com (Y.L.); leizhenni@mail.nwpu.edu.cn (Z.L.); gpf03@126.com (Y.C.); zhanmei@nwpu.edu.cn (M.Z.); 2 School of Mechanics, Civil Engineering and Architecture, Northwestern Polytechnical University, Xi’an 710129, China

**Keywords:** titanium alloy, tri-modal microstructure, strain heterogeneity, strain localization

## Abstract

Grain-scale strain heterogeneity characteristics play a critical role in the ductile damage behavior and mechanical properties of two-phase titanium alloys. In this work, the grain-scale strain distribution, strain heterogeneity, and strain localization of titanium alloy with tri-modal microstructure (consisting of equiaxed α (α_p_), lamellar α (α_l_), and β transformed matrix (β_t_)) during tensile deformation were experimentally investigated. The results show that the strain probability distribution of the whole microstructure obeys normal distribution during deformation. Significant strain heterogeneities exist in each constituent (α_p_, α_l_, and β_t_) and the whole microstructure. At lower macro-strain, α_p_ and α_l_ exhibit higher average strain than those of β_t_ and the whole of the microstructure. Meanwhile, strain heterogeneity of each constituent is small and has a negligible change. The strain heterogeneity of the whole microstructure is mainly determined by α_p_. At larger macro-strain, some highly deformed regions produce and their positions do not change during further deformation. As a result, the strain heterogeneity of each constituent increases fast, and the strain heterogeneity of whole microstructure is mainly related to α_l_ in this deformation stage. On the other hand, two types of strain localization may be generated within α_p_ and α_l_ and at the α_p_/β_t_ and α_l_/β_t_ boundaries, respectively. The former type is caused by transgranular intense slip deformation and presents crystal orientation dependence. The latter type is related to the boundary sliding and presents spatial distribution dependence for α_l_. These strain localizations greatly determine the micro-damages, thus forming the corresponding micro-voids within α_p_ and α_l_ and the micro-cracks at α_p_/β_t_ and α_l_/β_t_ boundaries in tri-modal microstructure at larger deformation.

## 1. Introduction

Two-phase titanium alloys are widely used in medicine and aviation fields. In the field of medical engineering, they are mainly applied in the form of orthopedic implants and surgical implants, such as the hip implants, knee joints, and substitutions of shoulder, spine, elbow, and hand. In the aviation field, the typical specific application examples include the vane frame, fan disk, landing gears, etc., where excellent microstructure and mechanical properties are required [1,2,3,4,5,6]. Due to the different crystal structures and constituent behavior between constituent phases (the hexagonal close-packed α and the body-centered cubic β), deformations in two-phase titanium alloys are always heterogeneous, which can be characterized by strain heterogeneity [7,8]. Such heterogeneity may lead to incompatible deformation, even strain localization and shear bands, dramatically determining the ductile damage and mechanical properties [9,10,11]. From a microstructural point of view, morphology, volume fraction, size, crystal orientation, and spatial arrangement of each constituent phase all significantly affect the heterogeneity. Therefore, strain heterogeneity is a critical grain-scale phenomenon bridging microstructures and mechanical properties. Deep understanding on strain heterogeneity is essential for optimizing the microstructure and mechanical properties of titanium alloy.

Recently, many experimental and micromechanical modeling investigations have been conducted on the characteristic of grain-level strain heterogeneity and damage behavior of two-phase metals, especially the dual phase (DP) steel. Ghadbeigi et al. [12] studied the local deformation and damage mechanisms of DP600 steel by conducting in-situ deformation and microscopic digital image correlation (DIC). They found that the strain within ferrite phase is much larger than that within martensite, and severe deformation localization often produces within ferrite grains. The failure of martensite occurs mainly due to the micro-crack initiation from boundaries with ferrite, while the failure of the ferrite matrix mainly occurs by void formation within ferrite. By the combination of in-situ experiment and micromechanical simulation coupling damage model, Matsuno et al. [9] found that the martensite fracture and interface decohesion both play great roles in the strain partitioning, strain localization, and the subsequent void growth and coalescence for DP steel. Lai et al. [13] and Jafari et al. [14] investigated the influence of martensite volume fraction and spatial distribution on the strain localization and damage behavior of DP steel, respectively. These works suggest that the strain heterogeneities and failure mechanism of DP steel strongly depend on the morphology, content, size, and distribution of ferrite and martensite.

For two-phase titanium alloy, the morphology, spatial distribution, and disparity of constitutive behavior of constituent phases (α and β phases) are different from those of DP steel (ferrite and martensite), which make it present different rules on the strain heterogeneities and damage behavior. Barkia et al. [15] experimentally investigated the evolution law of strain heterogeneity and formation of highly deformed band in the tensile of commercial purity (CP) titanium. Through the high-resolution digital image correlation, Lunt et al. [16] quantified the strain distribution in both equiaxed α and fine α_2_ precipitation in the deformation of Ti-64 alloy with equiaxed microstructure. Ji and Yang [17] developed a microstructure-based finite element model to analyze the strain distribution of TA15 alloy with bimodal microstructure under tensile loading, which was found greatly dependent on the volume fraction, spatial distribution, and yield stress of each constituent. Zhang et al. [18] simulated the grain-scale strain heterogeneity and discussed its role in the shear band and vertical split failure during tensile deformation of Ti-64 alloy with equiaxed and bimodal microstructures. Katani et al. [19] developed a micromechanical model coupling the Gurson-Tvergaard-Needleman damage model, by which they predicted the effect of microstructure morphology on strain localization and void nucleation during tensile deformation of Ti-64 alloy with equiaxed microstructure. The informed works mainly focus on the strain heterogeneities of equiaxed and bimodal microstructure, but there is little concern on the tri-modal microstructure consisting of equiaxed α (α_p_), lamellar α (α_l_), and β transformed matrix (β_t_). It has been reported that tri-modal microstructure is a potential microstructure type presenting better combination of strength, fracture toughness, and fatigue property [20]. The more complex constituents and morphology in tri-modal microstructure may lead to more complicated grain-scale incompatible deformation. Therefore, revealing the characteristics of grain-scale strain heterogeneity in tri-modal microstructure is needed.

In this paper, the grain-scale strain distribution of titanium alloy with tri-modal microstructure during tensile deformation was characterized by microscopic DIC technique. Then, the strain heterogeneity characteristics, strain localization mechanism, and its effect on the micro-damage were analyzed. It will deepen the understanding on grain-scale strain heterogeneity and damage behavior of titanium alloy with tri-modal microstructure.

## 2. Experimental Procedure

### 2.1. Material and Initial Microstructure

**Figure 1:**
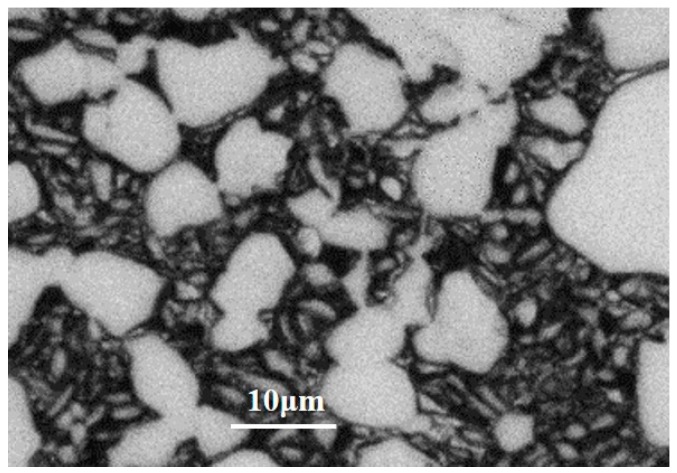
Initial microstructure of the as-received wrought TA15 alloy.

**Figure 2:**
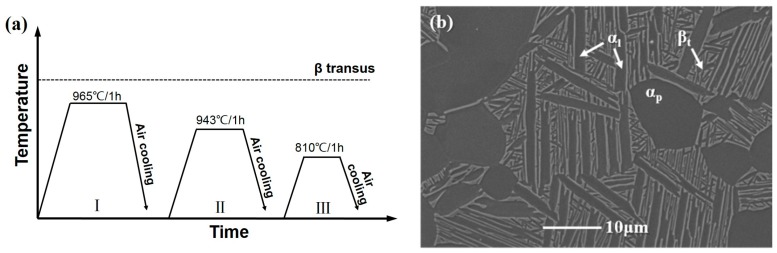
Processing schedule for obtaining tri-modal microstructure (**a**) and the obtained tri-modal microstructure (**b**).

The material used in this work is wrought TA15 titanium alloy provided by Western Superconducting Technologies Co., Ltd. (Xi’an, China). Its chemical compositions (wt%) are as follows: Al: 6.69; Mo: 1.77; V: 2.25; Zr: 2.26; Fe: 0.14; and Ti balance. The measured β-transus temperature is 985 °C. The initial microstructure is a typical equiaxed microstructure, as shown in Figure 1. To obtain a tri-modal microstructure, the processing schedule shown in Figure 2a is employed. The formation mechanism of tri-modal microstructure under this processing schedule has been reported in the author’s previous work in detail [7,21]. Figure 2b shows the obtained tri-modal microstructure, which consists of about 22.6% α_p_, 21.6% α_l_, and β_t_ balance. The most striking feature of a tri-modal microstructure is the existence of α_l_ compared to equiaxed and bimodal microstructures.

### 2.2. Tensile Testing and Mapping of Strain Field

**Figure 3:**
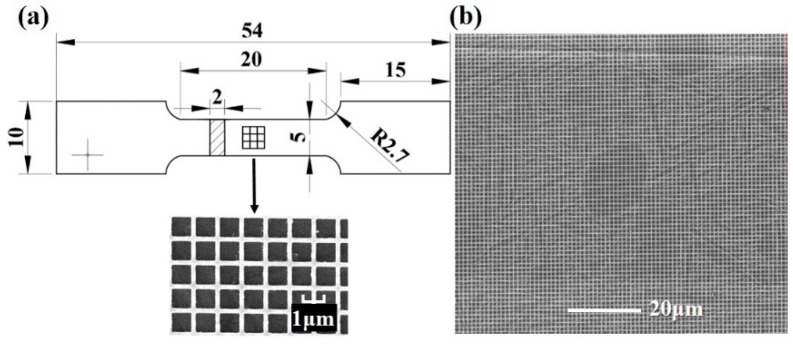
Schematic of the geometry and micro-grid of the tensile specimen (**a**) and area of interest (AOI) (**b**). The unit of dimension of sample is mm.

The processed material with tri-modal microstructure was machined to a plate tensile specimen. The detailed geometry and dimensions are shown in Figure 3a. The lengths of whole sample, parallel section, and grasp sections are 54, 20, and 15 mm, respectively. The widths of a parallel section and grasp section are 10 and 5 mm, respectively. The thickness of the sample is 2 mm. The fillet between parallel and grasp sections is 2.7 mm. Before the tensile test, the specimen surface was mechanically and electrolytically polished in a solution of 5% HClO_4_ + 65% CH_3_OH + 40% C_4_H_10_O for 40 s firstly. The electron beam lithography technique was then employed to print a gold micro-grid with the area of 1 × 1 mm^2^ at the center (Figure 3a), which wasconducted on a CABL-9000C electron beam lithography system (Crestec corporation, Tokyo, Japan) equipped with the Nanometer Pattern Generation System. The detailed introduction for this technique can be found in [22]. In this work, the line width and mesh size of micro-grid are 0.2 and 1 μm, respectively. After that, the specimen was intermittently stretched to four different engineering macro-strains using a Bairoe tensile testing machine (Bairoe, Shanghai, China) with the strain rate of 0.0001 s^−1^ at room temperature. The macro-strains were detected by laser extensometer (Epsilon Technology Corp, Jackson, MS, USA) as 1.45%, 2.77%, 4.7%, and 12.75%, respectively. After each tensile stage, the micro-grid in the area of interest (AOI) (Figure 3b) was observed by a scanning electron microscope (Carl Zeiss AG, Oberkochen, Germany).

Subsequently, the strain field in AOI was calculated by correlating the images of deformed grids with the reference image of undeformed state at different macro-strains. The detailed calculating methods are as follows [23]. First, each square in micro-grid was divided into four triangles by two diagonal lines. For each triangle, the coordinates of vertices before and after deformation are defined as $\left(x_{0}^{1},y_{0}^{1}),\;(x_{0}^{2},y_{0}^{2}),\;(x_{0}^{3},y_{0}^{3}\right)$ and $\left(x_{}^{1},y_{}^{1}),\;(x_{}^{2},y_{}^{2}),\;(x_{}^{3},y_{}^{3}\right)$, respectively. The displacement components of each vertex after deformation are

(1) $$\begin{array}{l} u^{1}=x^{1}-x_{0}^{1},v^{1}=y^{1}-y_{0}^{1}\\ u^{2}=x^{2}-x_{0}^{2},\;v^{2}=y^{2}-y_{0}^{2}\\ u^{3}=x^{3}-x_{0}^{3},\;v^{3}=y^{3}-y_{0}^{3} \end{array}$$

The displacement $\left(u^{i},v^{i}\right)$ of any interior point $\left(x^{i},y^{i}\right)$ in each triangle can be represented by Equation (2) based on the liner relationship between them.

(2) $$\begin{array}{l} u^{i}=a+bx^{i}+cy^{i}\\ v^{i}=d+ex^{i}+fy^{i} \end{array}$$

The values of *a*, *b*, *c*, *d*, *e*, and *f* in Equation (2) can be calculated by substituting Equation (1) into Equation (2). The Green-Lagrange strain in two dimensions can be represented by the differential forms of Equation (3):

(3) $$\begin{array}{l} \varepsilon_{xx}=\frac{1}{2}(\frac{\partial u^{i}}{\partial x}\frac{\partial u^{i}}{\partial x}+\frac{\partial v^{i}}{\partial x}\frac{\partial v^{i}}{\partial x})\\ \varepsilon_{yy}=\frac{1}{2}(\frac{\partial u^{i}}{\partial y}\frac{\partial u^{i}}{\partial y}+\frac{\partial v^{i}}{\partial y}\frac{\partial v^{i}}{\partial y})\\ \gamma_{xy}=\frac{1}{2}(\frac{\partial u^{i}}{\partial x}\frac{\partial u^{i}}{\partial y}+\frac{\partial v^{i}}{\partial x}\frac{\partial v^{i}}{\partial y})\\ \varepsilon_{eq}=\sqrt{\frac{4}{9}(\varepsilon_{x}^{2}-\varepsilon_{x}\varepsilon_{y}+\varepsilon_{y}^{2})+\frac{4}{3}\gamma_{xy}^{2}} \end{array}$$

where $\varepsilon_{i}(i=xx,\;yy),\;\gamma_{xy}\;\mathrm{and}\;\varepsilon_{eq}$ are the average of the total normal strain, the shear strain, and the equivalent strain of each triangle, respectively. Finally, the strain of each square can be obtained by averaging the strains of four triangles within it. The calculation results show that the distributions of axial strain and equivalent strain are very close for all macro-strains due to the unidirectional tensile deformation. Here, the axial strain field in AOI was used to analyze the strain heterogeneity characteristics of the tri-modal microstructure. Moreover, the microstructure graph was superimposed on the strain map to analyze the dependence of strain distribution on microstructure.

## 3. Results and Discussion

### 3.1. Characteristics of Strain Heterogeneity

**Figure 4:**
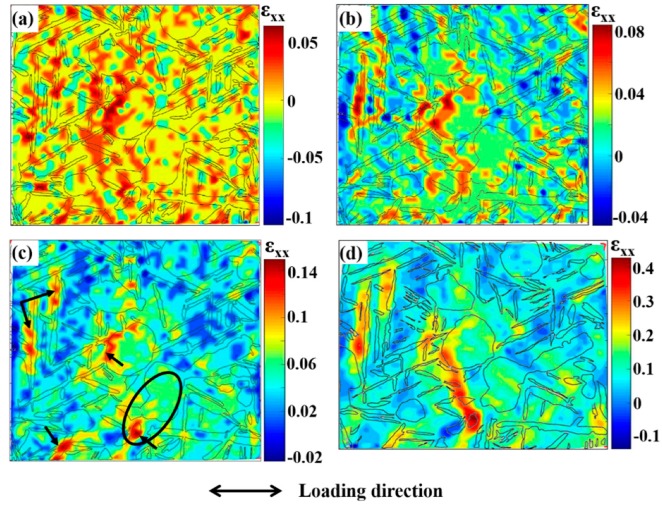
Axial strain maps at different tensile macro-strains: (**a**) 1.45%; (**b**) 2.77%; (**c**) 4.7%; (**d**) 12.75%.

Figure 4 shows the evolution of the axial strain field in the AOI of tri-modal microstructure during tensile tests. From Figure 4a, it can be found that the strain in the whole region is small and distributes relatively uniformly at the early stage of deformation. No obvious strain localization region can be found. As the deformation proceeds, the overall strain level increases a little and the strain starts to preferentially accumulate at some regions, as shown in Figure 4b. When the macro-strain reaches to 4.7%, four highly deformed regions are formed, as indicated by arrows in Figure 4c. They are located within α_p_ and α_l_ and at the α_p_/β_t_ and α_l_/β_t_ boundaries, respectively. At the subsequent deformation, these highly deformed regions nearly keep in the same locations, while the strain level increases a substantial amount (Figure 4d). The corresponding strain localization mechanisms for four highly deformed regions will be explained in Section 3.2 in detail.

**Figure 5:**
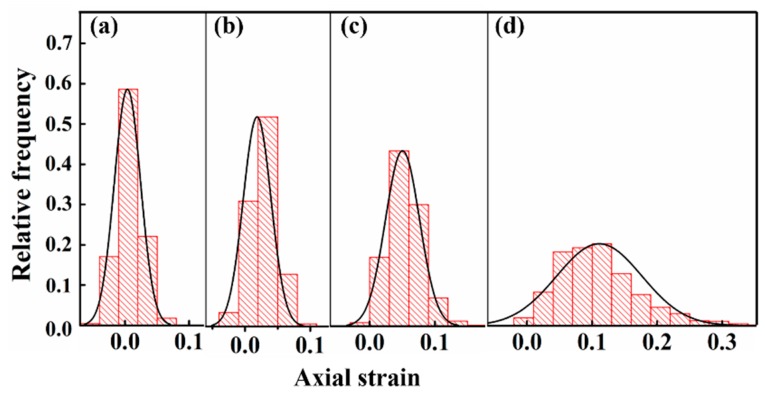
Variation of probability distributions of axial strain with tensile macro-strain: (**a**) 1.45%; (**b**) 2.77%; (**c**) 4.7%; (**d**) 12.75%.

To quantitatively describe the strain distribution characteristics, the probability distribution of strains at grid nodes under different macro-strains are shown in Figure 5. Here, the relative frequency is used to represent the probability of the strain of each point locating in a certain strain range. It is calculated by the following equation:

(4) $$f_{i}\;=\;N_{i}/N_{total}$$

where *N_i_* is the amount of points locating in the strain Range *i*, and *N_total_* is the total points of the domain. It can be found that the probability distribution of strain at different deformation stages all obey normal distribution (as fitted by black lines). This phenomenon is the same as the normal distribution of strain in CP titanium [15] and magnesium alloy [22], while different to the lognormal distribution of strain in pearlite microstructure [23]. In addition, we can see that the fitted normal distribution curve widens as the deformation proceeds. This indicates that both the strain heterogeneity and average strain of the tri-modal microstructure increase as deformation occurs.

**Figure 6:**
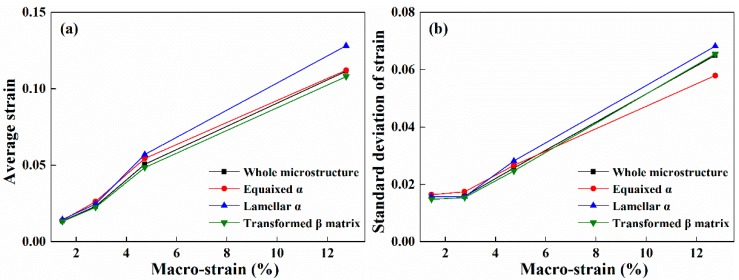
Variations of average (**a**) and standard deviation (**b**) of strain in the whole microstructure and different constituents.

Figure 6 shows the variations of average and standard deviation (STD) of strain in the whole microstructure (WM) and each constituent (α_p_, α_l_, and β_t_). Here, STD is an important index that can evaluate the strain heterogeneity. Overall, there exist two change stages with different rules for both the average and STD of the strain in the whole deformation process. At lower macro-strain (<4.7%), the average strains of α_p_ and α_l_ are close and slightly larger than WM and β_t_, as shown in Figure 6a. Lei et al. [7] have experimentally investigated the nano-hardness of constituents in tri-modal microstructure and found that the α_p_ and α_l_ are softer than β_t_ at room temperature. Thus, α_p_ and α_l_ are easier to deform than β_t_ and present a larger average strain. Meanwhile, STD of WM and each constituent are all relatively small and present negligible variations in this deformation stage. It is worth noting that STD of α_p_ is much bigger than other constituents, which suggests that α_p_ is mainly responsible for the strain heterogeneity of WM in this deformation stage. At larger macro-strain (≥4.7%), both the average strain and STD of each constituent and WM increase quickly. Moreover, it can be found from Figure 6a,b that the average strain and STD of α_l_ are both obviously larger than other constituents, which is related to the formation of strain localization regions near α_l_ (see Figure 4c,d). On the contrary, α_p_ presents the smallest STD and strain heterogeneity, which may be related to its features of easy-to-deform and superior deformation compatibility. The above results indicate that α_l_ plays a more important role in the strain heterogeneity of WM at larger macro-strain (≥4.7%). It can be concluded that the softer α_p_ and α_l_ in tri-modal microstructure undertakes more deformation and greatly affects the strain heterogeneity in tri-modal microstructure. Similarly, Ji and Yang [17] have found that the strain significantly localizes in softer primary α phase and contribute to the strain heterogeneity in TA15 alloy with bimodal microstructure.

### 3.2. Strain Localization and Micro-Damage

As mentioned above, four highly deformed regions, i.e., strain localization region, are formed when the macro-strain reaches to 4.7% (Figure 4c). Their formations play great roles in the strain heterogeneity and micro-damage behavior. Thus, in this section, the strain localization mechanisms are discussed by analyzing the corresponding characteristics of grid changes and microstructure morphology after deformation. Moreover, their relations to the micro-damage behavior are also analyzed.

**Figure 7:**
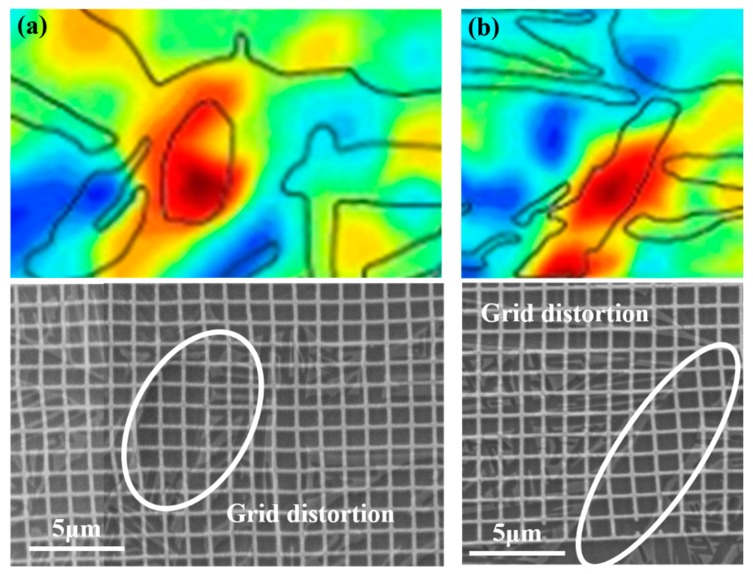
The typical strain localization regions and corresponding grid change within equiaxed α (**a**) and within lamellar α (**b**) at macro-strain of 4.7%.

**Figure 8:**
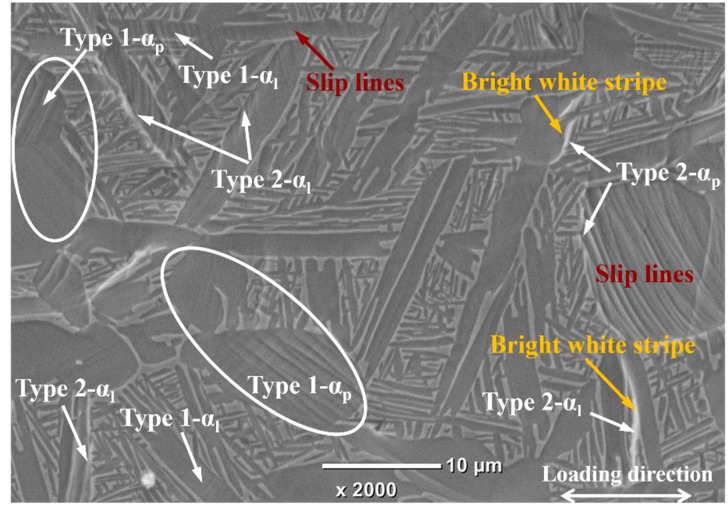
The morphology of deformed tri-modal microstructure after tensile deformation (the sample surface was mechanically and electrolytically polished before tensile deformation).

According to the location of strain localization regions (indicated by arrows in Figure 4c), these regions can be classified into two types: Type 1, strain localization within α_p_ and α_l_; Type 2, strain localization at the α_p_/β_t_ and α_l_/β_t_ boundaries. Figure 7 shows the local strain distribution and grid change of Type 1 strain localization regions. It can be found that the strain localization corresponds to the obvious grid distortion within α_p_ and α_l_, as indicated by the ellipses. Meanwhile, quantities of slip lines are found within some α_p_ and α_l_ in the surface of deformed microstructure, as shown in Figure 8. These suggest that the dislocation slip is the main deformation mode within α_p_ and α_l_; moreover, intense slip deformation within some grains will generate local strain localization (Type 1). Some potential Type 1 strain localization are indicated in Figure 8. We can find that intense slip deformation and Type 1 strain localization only occurs in some particular α_p_ and α_l_ grains. Even for some adjacent grains, only individual grains will produce strain localization. For example, there are two adjacent α_p_ grains in each ellipse in Figure 4c and Figure 8; however, only one of them produces intense slip deformation and strain localization after deformation. This is because the slip deformation within α_p_ and α_l_ are strongly dependent on the crystal orientation, which are easier to take place in soft-oriented α_p_ and α_l_. Thus, the neighboring grains may present different degrees of slip deformation and strain localization. These indicate that Type 1 strain localization within α_p_ and α_l_ present crystal orientation dependence.

**Figure 9:**
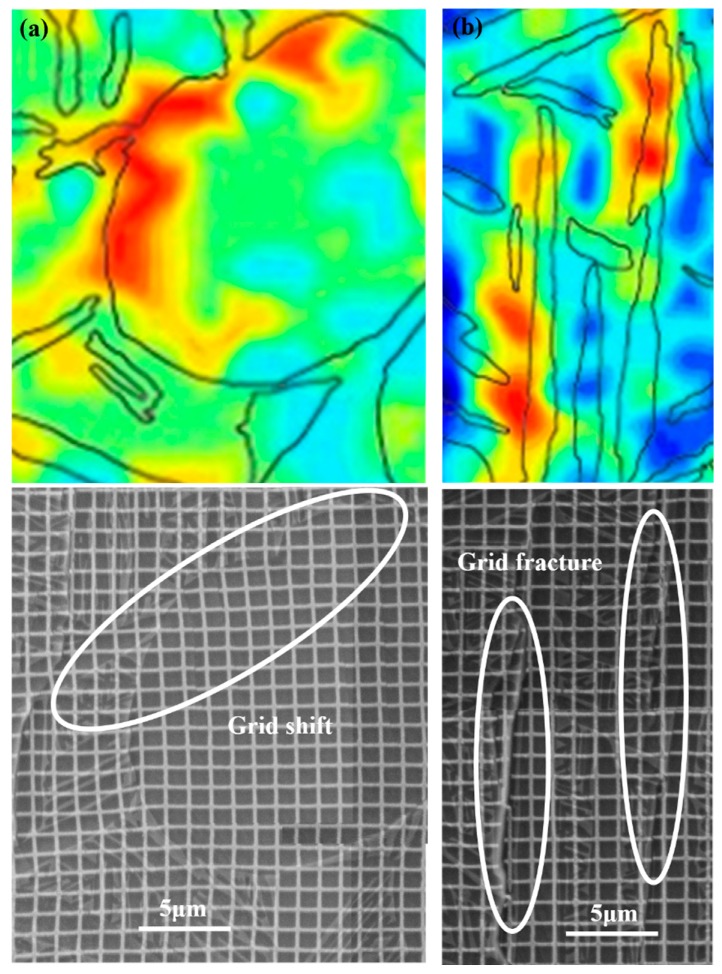
The typical strain localization regions and corresponding grid change at the boundary of α_p_/β_t_ (**a**) and the boundary of α_l_/β_t_ (**b**) at macro-strain of 4.7%.

As for Type 2 strain localization, the corresponding strain distribution and grid change are given in Figure 9. Obvious shift and fracture of microgrids are found at the boundaries of α_p_/β_t_ and α_l_/β_t_, which implies the boundary sliding occurs in these regions. Barkia et al. [15] have also observed the boundary sliding at α_p_/α_p_ boundary during the tensile deformation of CP titanium at room temperature. Similarly, Katani et al. [19] have also reported the shear deformation and fracture along α_p_/β_t_ boundary during the tensile deformation of Ti-64 alloy at room temperature. The corresponding mechanism can be inferred from the corresponding features of deformed microstructure (Figure 8) as follows. Intense slip deformation is activated in the margin of softer α_p_ and α_l_, while the neighboring β_t_ is relatively hard to deform. Dislocations pile up at grain boundaries and some of them are absorbed by grain boundaries. The absorbed dislocations will slip along interface under shear stress, which then lead to the boundary sliding and shows the bright white stripe (indicated by yellow arrows in Figure 8). On the other hand, it can be found that the included angles between the α_l_ producing Type 2 strain localization and loading axis all range within 60–90° (Figure 4 and Figure 8). This indicates that Type 2 strain localization presents spatial distribution dependence, which may be mainly related to the lamellar morphology of α_l_.

**Figure 10:**
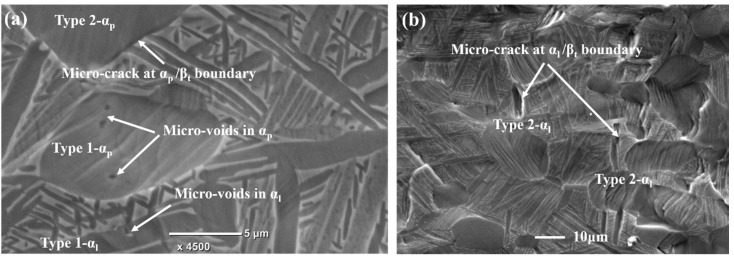
The micro-damage features of tri-modal microstructure after larger tensile deformation: (**a**) micro-voids within α_p_ and α_l_ and micro-crack at α_p_/β_t_ boundary; (**b**) micro-crack at α_l_/β_t_ boundary (the sample surface was mechanically and electrolytically polished before tensile deformation).

Figure 10 shows the typical micro-damage features of tri-modal microstructure after larger tensile deformation. According to the damage feature and location, they can also be classified into two typical types: the micro-voids within α_p_ and α_l_ and the micro-cracks at α_p_/β_t_ and α_l_/β_t_ boundaries, as indicated in Figure 10. It can be found that the α_p_ and α_l_ grains producing micro-voids all present intense slip deformation, which is right the deformation feature of Type 1 strain localization. This suggests that the formation of micro-voids within α_p_ and α_l_ is closely related to Type 1 strain localization mentioned above. On the other hand, the locations of micro-cracks at α_p_/β_t_ and α_l_/β_t_ boundaries right correspond to the above Type 2 strain localization, which are formed due to the boundary sliding. Thus, it can be concluded that the strain localization plays decisive roles in the formation and features of micro-damage in the tri-modal microstructure of titanium alloy.

Above, it was found that significant grain-scale strain heterogeneities exist in each constituent (α_p_, α_l_, and β_t_) and whole microstructure of the tri-modal microstructure during tensile deformation. Moreover, two types of strain localization may be formed due to the intense slip deformation and boundary sliding. This will then greatly determine the micro-damage behavior. It was found that the unique lamellar α in the tri-modal microstructure has a great effect on the strain heterogeneity and strain localization. As mentioned in Section 2.1, the most striking feature of the tri-modal microstructure is the existence of α_l_ compared to bimodal microstructures. Thus, the effect of α_l_ on deformation behavior was briefly discussed here. As for the micro-scale strain distribution, the α_l_ presents close average strain but lower STD than α_p_ at lower macro-strain (<4.7%), while the α_l_ presents a much larger average strain and STD than both α_p_ and β_t_ at larger macro-strain (≥4.7%). These suggest that α_l_ plays an important role in the strain heterogeneity of the whole microstructure at larger macro-strain (≥4.7%). As far as the strain localization is concerned, strain localization can be generated at the interior and boundary of α_l_. The strain localization at α_l_ boundary presents spatial distribution dependence. These strain localization characteristics related to α_l_ play great roles in the micro-damage behavior of the tri-modal microstructure. Thus, it is important to control the content and spatial distribution of α_l_ to optimize the tri-modal microstructure and corresponding mechanical properties. However, it is difficult to thoroughly investigate the effects of morphology, volume fraction, size, crystal orientation, and spatial arrangement of each constituent phase on the strain heterogeneity of the tri-modal microstructure by only the experiment method. It is greatly limited by the difficulties in the microstructure morphology tailor, orientation characterization, strain distribution evaluation, deformation mode analysis, and so on. Thus, further micromechanical modeling investigation considering the real microstructure, crystal orientation, slip and damage behavior of each constituent, properties of grain and phase boundaries should be conducted to deepen the understanding of grain-scale deformation and damage behavior in the tri-modal microstructure in the future.

## 4. Conclusions

In this paper, the grain-scale strain heterogeneity characteristics, strain localization mechanism and its effect on the micro-damage during tensile deformation of titanium alloy with tri-modal microstructure were experimentally investigated. The following conclusions can be drawn:

(1) The strain probability distribution of the whole tri-modal microstructure obeys a normal distribution during tensile deformation. Significant grain-scale strain heterogeneities exist in each constituent (α_p_, α_l_, and β_t_) and the whole microstructure. In addition, some highly deformed regions form at a higher macro-strain and keep the same positions during further deformation.

(2) At lower macro-strain (<4.7%), α_p_ and α_l_ present bigger average strain than β_t_ and the whole microstructure, while the strain heterogeneity of each constituent is small and has negligible change. The strain heterogeneity of the whole microstructure is mainly determined by α_p_. At larger macro-strain (≥4.7%), the strain heterogeneity of each constituent increases fast, and the strain heterogeneity of whole microstructure is mainly related to α_l_.

(3) There are two typical types of strain localization regions produced in the tri-modal microstructure. The first type is within α_p_ and α_l_, which is caused by the transgranular intense slip deformation and presents crystal orientation dependence. The second type is at the α_p_/β_t_ and α_l_/β_t_ boundaries, which is related to the boundary sliding caused by strain incompatibility. In addition, the second type related to α_l_ presents spatial distribution dependence, which mainly occurs at the α_l_ presenting the included angles of 60–90° with respect to loading axis. These strain localizations greatly determine the micro-damage behavior, thus producing the corresponding micro-voids within α_p_ and α_l_ and the micro-cracks at α_p_/β_t_ and α_l_/β_t_ boundaries in tri-modal microstructure after larger deformation.
